# White matter of perinatally HIV infected older youths shows low frequency fluctuations that may reflect glial cycling

**DOI:** 10.1038/s41598-021-82587-5

**Published:** 2021-02-04

**Authors:** Manoj K. Sarma, Amrita Pal, Margaret A. Keller, Tamara Welikson, Joseph Ventura, David E. Michalik, Karin Nielsen-Saines, Jaime Deville, Andrea Kovacs, Eva Operskalski, Joseph A. Church, Paul M. Macey, Bharat Biswal, M. Albert Thomas

**Affiliations:** 1 Department of Radiological Sciences, David Geffen School of Medicine at UCLA, 10833 Le Conte Avenue, Los Angeles, CA 90095-1721 USA; 2 UCLA School of Nursing, University of California, Los Angeles, Los Angeles, CA USA; 3 Pediatrics, Harbor-UCLA Medical Center, Torrance, CA USA; 4 The Lundquist Institute for Biomedical Innovation at Harbor-UCLA Medical Center, Torrance, CA USA; 5 Psychiatry and Biobehavioral Sciences, David Geffen School of Medicine at UCLA, Los Angeles, CA USA; 6 Infectious Diseases-Pediatrics, Miller Children’s Hospital of Long Beach, Long Beach, CA USA; 7 David Geffen School of Medicine at UCLA, Los Angeles, CA USA; 8 Keck School of Medicine of University of Southern California, Los Angeles, CA USA; 9 Los Angeles+USC Medical Center, Los Angeles, CA USA; 10 Pediatrics, Keck School of Medicine of University of Southern California, Children’s Hospital Los Angeles, Los Angeles, CA USA; 11 Biomedical Engineering, New Jersey Institute of Technology, Newark, NJ USA

**Keywords:** Biomarkers, Paediatric research, HIV infections

## Abstract

In perinatally HIV-infected (PHIV) children, neurodevelopment occurs in the presence of HIV-infection, and even with combination antiretroviral therapy (cART) the brain can be a reservoir for latent HIV. Consequently, patients often demonstrate long-term cognitive deficits and developmental delay, which may be reflected in altered functional brain activity. Our objective was to examine brain function in PHIV on cART by quantifying the amplitude of low frequency fluctuations (ALFF) and regional homogeneity (ReHo). Further, we studied ALFF and ReHo changes with neuropsychological performance and measures of immune health including CD4 count and viral loads in the HIV-infected youths. We found higher ALFF and ReHo in cerebral white matter in the medial orbital lobe for PHIV (*N* = 11, age mean ± sd = 22.5 ± 2.9 years) compared to controls (*N* = 16, age = 22.5 ± 3.0 years), with age and gender as co-variates. Bilateral cerebral white matter showed increased spontaneous regional activity in PHIV compared to healthy controls. No brain regions showed lower ALFF or ReHo in PHIV compared to controls. Higher log10 viral load was associated with higher ALFF and ReHo in PHIV in bilateral cerebral white matter and right cerebral white matter respectively after masking the outcomes intrinsic to the brain regions that showed significantly higher ALFF and ReHo in the PHIV compared to the control. Reductions in social cognition and abstract thinking in PHIV were correlated with higher ALFF at the left cerebral white matter in the left medial orbital gyrus and higher ReHo at the right cerebral white matter in the PHIV patients. Although neuroinflammation and associated neuro repair were not directly measured, the findings support their potential role in PHIV impacting neurodevelopment and cognition.

## Introduction

Although there is still no definitive cure for human immunodeficiency virus (HIV), the tremendous success of combination antiretroviral therapy (cART) has transformed both perinatal HIV (PHIV) and HIV into a treatable chronic disease^1–5^. However, it has been observed that poor penetration of some antiretrovirals across the blood–brain barrier may provide insufficient protection of the central nervous system (CNS)^6–8^. This has led to serious concerns regarding the brain’s role as a sanctuary site for HIV. It has been reported that long-term cART treatment may be associated with potential mitochondrial toxicity, metabolic abnormalities, impaired neurogenesis and may cause neuronal loss^9–11^. Furthermore, the many children who have survived to adulthood from earlier eras with less efficacious regimens may experience indolent ongoing brain injury. Consequently, although increasing numbers of children born with HIV infection are surviving into adulthood, they remain at risk for long-term central nervous system damage^12–17^.

Neurodevelopment takes place in HIV youths in the presence of HIV-infection as PHIV patients acquired the infection at birth and in utero. On the other hand, in adult-acquired HIV infection, neuro-development may more likely have happened prior to infection. As a result, brain related effects of chronic HIV-infection may vary in PHIV individuals compared to other HIV-infected patients. Developmental delay and behavioral problems have been reported from neuropsychological studies of PHIV-infected children receiving ART^18,19^. In addition, deficiency in neurocognitive functions including psychomotor ability, language, executive function, visual–spatial, and memory^12,13,15,20–25^ has been reported compared to uninfected healthy controls. Our recent study on perinatally HIV-infected older youths receiving ART^26^ showed a decrease in attention/processing speed in HIV-infected youths relative to HIV-negative controls, indicating that cognitive abnormalities persist as these children reach adolescence and adulthood. Brain abnormalities likely underlie these cognitive and other developmental difficulties in HIV-infected youths.

Previous neuroimaging findings in perinatally HIV-infected children on ART include ventricular enlargement and/or sulcal widening, calcification of the basal ganglia and corpus callosum, white matter signal abnormalities and lesions, reduced white matter, and decreased white matter integrity^15,27–37^. Further, some of these studies have shown that clinical, immunologic, and virologic measures were associated with volumetric measures, diffusivity markers, shape deformation, and WM alterations^15,32–34,38,39^.

Brain function as well as structure is likely affected in HIV-infected youths. Brain function can be studied with resting-state functional magnetic resonance imaging (rs-fMRI)^40^. Resting fMRI measures the spontaneous blood oxygen level-dependent (BOLD) signal, which reflects underlying neural activity, and which is used to evaluate regional interactions, and functional connectivity (FC) between brain networks. Resting fMRI avoids performance confounds of task-based imaging making it more suitable for patients with disorders of consciousness, potentially impaired clinical subjects and pediatric populations. Another advantage of rs-fMRI over task-based fMRI is the ability to identify many spatially distinct brain networks simultaneously. It has provided significant insights on brain development^41,42^ and has emerged as an interesting biomarker for measuring connectivity within brain networks in multiple conditions including brain tumors and psychiatric disorders such as schizophrenia^43–53^. Regional homogeneity (ReHo) is one rs-fMRI metric that reflects synchrony of adjacent regions, and is considered a marker of local functional organization^54,55^. Another rs-fMRI technique measures the low frequency fluctuations of the blood oxygen level dependent BOLD signal within the frequency range (0.01–0.08 Hz); this measure is termed Amplitude of Low Frequency Fluctuations (ALFF)^55^. The ALFF measure has been related to neural activity, but may reflect other phenomena as well, including astrocyte activity^55–58^. The ALFF measure may therefore relate indirectly to the inflammatory state of the brain, and hence is relevant to HIV patients who likely have an ongoing high inflammatory state. The fractional ALFF (f-ALFF) analyses normalize the ALFF power by dividing by the total power in the entire detectable frequency range to represent the relative contribution of low frequency oscillations.

Although rs-fMRI has been examined in many diseases, there are limited studies on brain connectivity alteration in HIV-infected patients^59–65^ and their correlation with neurocognitive impairment. In adult HIV-infected patients, rs-fMRI studies reported altered FC within different brain networks, including lateral occipital cortex (LOC)^65^, salience, executive control, and default mode (DM) networks^64^. Both lower and higher internetwork correlations^64^, unusual functional connectivity between the dorsal caudate and the dorsolateral prefrontal cortex^60^ and connection between HIV and measures of centrality difference^63^ have also been obesrved. On the other hand, Janssen et al.^61^ did not observe differences in subcortical connectivity between healthy controls and virologically controlled HIV-infected adult patients who were otherwise healthy. Compared to HIV-negative controls, Ortega et al.^62^ found lower cortico-striatal functional connectivity in HIV-infected patients between the striatum and the default mode network and ventral attention network. They also observed that virologically controlled HIV-infected patients showed higher connectivity between these networks than patients not virologically controlled. In HIV-associated neurocognitive disorder groups, reduced synchronicity in the salience and executive networks despite viral suppression was reported by Chaganti et al.^59^.

To date, there are only a smaller number of rs-fMRI studies in PHIV children receiving ART^56,66,67^. In their study on PHIV youth receiving ART, Herting et al. observed global alterations in the “default mode network” (DMN), with significant associations between disease severity and lower connectivity within the DMN^56^. Furthermore, they found that patterns of connectivity with the posterior cingulate cortex (PCC) and medial prefrontal cortex (mPFC) varied as a function of peak HIV RNA and the rs-fMRI patterns predicted processing speed ability. Toich et al.^66^ examined the effects of HIV infection on FC in 7-year-old children who had received early ART treatment. They observed reduced long-range connectivity and increased short-range connectivity suggesting developmental delay. During infancy, they also found that poor immune health, as reflected by either lower CD4 or CD4% at enrollment, was associated with localized FC increases in the somatosensory, salience and basal ganglia networks and summarized that HIV may affect brain development from its earliest stages and persist into childhood, despite early ART. Yadav et al.^67^ evaluated the functional brain activity in HIV-infected children (mean age 9.3 years) by ALFF and FC. Compared with controls, the HIV-group showed lower ALFF in the left middle temporal gyrus, precentral and post central gyrus (principally gray matter regions), and altered FC between multiple brain regions. They also observed significantly lower NP scores in various domains, with scores correlated to ALFF and FC in HIV-infected children.

Although brain involvement with HIV is well documented for PHIV-infected infants and children, long-term neurologic outcomes for older HIV-infected youths are less understood. Herting et al. focused on the DMN connectivity in a sample with mean age of 16.5 years, Toich et al. looked at children around age 7, and Yadav et al. age 9^56,66,67^. We aimed to study an older age group. The objective of our current study was to examine whether youth (late teens and young adults) would show altered brain function as reflected in rs-fMRI changes compared with healthy controls. Since our earlier studies documented both white matter and gray matter changes in this population, we hypothesized that there would be differences in rs-fMRI in PHIV vs. control. Here we use rs-fMRI ALFF and ReHo changes to examine alterations in neuronal activity across the gray and white matter of the brain. Further, we studied the relationship of ALFF and ReHo changes with neuropsychological assessment results and measures of immune health such as CD4 count, viral loads in the HIV-infected youths. We hypothesized those alterations in rs-fMRI activity in PHIV infection would reflect changes in neuropsychological functioning.

## Materials and methods

### Participants/subjects

Eleven PHIV-infected youths (age 22.5 ± 2.9 years, range 19.6–29.1, 8 females) and sixteen healthy controls (HC) (age 22.5 ± 3.0 years, range 19.1–29.5, 9 females) participated in our study. The PHIV participants were recruited from four medical centers: Los Angeles County Harbor-UCLA Medical Center (Departments of Pediatrics and Medicine, Torrance, CA), Miller Children’s Hospital of Long Beach (Long Beach, CA), USC Medical Center’s Maternal, Child, and Adolescent Center for Infectious Diseases and Virology, and David Geffen School of Medicine at UCLA (Los Angeles, CA). The healthy controls were recruited from family members of the subjects, and through fliers at UCLA, the local junior college, the Lundquist Institute and neighboring communities. The research protocol was approved by the institutional review board (IRB) both at the Lundquist Institute for Biomedical Innovation at Harbor-UCLA Medical Center and at the University of California at Los Angeles. All methods were carried out in accordance with relevant guidelines and regulations, and followed the Health Insurance Portability and Accountability Act (HIPAA). All subjects completed study procedures voluntarily and signed informed consent. Participants were reimbursed for their time in the study.

### Study criteria

Study inclusion criteria were similar to our previous studies^26,31^ and consisted of the following: (1) 18–30 years of age; (2) perinatal acquisition of HIV or confirmation of HIV-uninfected status with Ora–Quick (OraSure Technologies, Bethlehem, PA 18015) buccal scraping (for HIV-subjects); (3) current treatment with combination antiretroviral medication for HIV-infected subjects; (4) post-menarchal status for all females since they were studied in the follicular phase of the menstrual cycle; (5) and for females negative urine pregnancy test on day of scanning. We excluded participants if they had: (1) a history of CNS opportunistic infection or other CNS condition (other than HIV); (2) severe metabolic disturbances, such as hepatic or renal failure; (3) metallic implants or braces or permanent retainers or other MRI exclusions; (4) claustrophobia; (5) Attention Deficit/Hyperactivity Disorder; (6) pregnancy (by interview and urine pregnancy test before scanning); (7) alcohol or other substance use/abuse including marijuana; (8) active psychiatric diagnosis; (9) use of chronic medication other than inhalers for asthma in control subjects; (10) severe school difficulties in control subjects; (11) female subjects pregnant or in luteal phase of menstrual cycle; (12) hepatitis C infection.

For HIV+ subjects, the following additional data were collected from chart review: age at first treatment for HIV, HIV viral load close to time of testing, highest known viral load, CD4 T cell counts close to time of testing, lowest known CD4, lowest known CD4%, current antiretroviral therapy, known presence of HIV encephalopathy, and history of maternal substance abuse during pregnancy. The clinical variables are summarized in Table 1 and demonstrate that these patients had been treated for many years with antiretroviral therapy. Data from a life time of HIV were not always complete and we have indicated in Table 1 such variables as the lowest known CD4 and CD4% and highest known viral load realizing that viral load was not a standard test when these patients were younger. We also note that we did not systematically assess adherence to medications and realize that adolescence is a time when patients can have poor compliance. Nonetheless, 54.5% of the study patients had an undetectable viral load. Of the 11 PHIV participants, four had a diagnosis of HIV encephalopathy while one patient was considered to have a probable diagnosis of HIV encephalopathy. Eight of these 11 patients had experienced school difficulties. In addition, two mothers had known substance abuse during pregnancy while information for one mother was not available, and for 8 there was no evidence of substance abuse during pregnancy.

**Table 1:** Demographic and clinical characteristics.

Characteristics	PHIV-infected youth (n = 11) Mean ± SD (range)	Healthy controls (n = 16)	p-value
Age (years)	22.5 ± 2.9	22.5 ± 3.0	0.96
Sex (male:female)	3 : 8	7:9	0.40
Age at ART initiation (months)	21.64 ± 36.96 (3–129)	–	–
Age at HIV diagnosis (months)	18.91 ± 36.75 (1–126)	–	–
Current CD4 T-cell count	506.73 ± 301.46 (55–963)	–	–
Lowest known CD4	268.82 ± 255.16 (37–635)	–	–
Lowest known CD4%	14 ± 10.39 (2–31)	–	–
Current Viral Load	13,124.82 ± 35,938.30 (20–11,9536)	–	–
Highest known viral load	383,283.91 ± 404,037.17 (18,475–1,223,892)	–	–
Current Log_10_ viral load	2.24 ± 1.40 (1–5.08)	–	–

Demographic and clinical characteristics of PHIV-infected youths and healthy controls. p-value shown for group differences assessed with independent samples t-test.

### MRI

All MRI studies were performed using a 3 T Prisma MRI scanner (Siemens Medical Solution, Erlangen, Germany), using a 16-channel phased-array head ‘receive’ coil. During data acquisition, subjects were instructed to stare at a spot in the scanner and remain awake. To minimize head movement, foam pads were placed on either side of the head. rs-fMRI scans were collected using an echo planar imaging (EPI) sequence with: TR/TE = 2000/27 ms, Flip angle = 90°, 40 slices, matrix size = 64 × 64; FOV = 240 × 240 mm^2^; acquisition voxel size = 3.75 × 3.75 × 4 mm^3^; and 180 volumes/scan. To facilitate EPI distortion correction, a field map was acquired before the rs-fMRI scan with: TR = 430 ms, TE = 7.35/9.81 ms, matrix size = 64 × 64, FOV = 192 mm, forty 4 mm slices, no gap. In addition, a high-resolution T_1_-weighted magnetization-prepared rapid gradient echo scan (MPRAGE) was acquired for anatomical information for better registration and overlay of brain activity. All the subjects were scanned at the same site.

### Neurocognitive data

Patients performed a neurocognitive battery test at a separate visit from the MRI data collection. These tests were assessed in depth separately, but were included here to aid with interpretation of significant findings. All subjects were administered a comprehensive neuropsychological assessment battery by a clinical psychology trainee in the following fixed order: MATRICS Consensus Cognitive Battery (MCCB)^68^ subtests include: Brief Assessment of Cognition in Schizophrenia (BACS): Symbol Coding, Category Fluency: Animal Naming, Trail Making Test: Part A (including Part B)^69^, Continuous Performance Test—Identical Pairs (CPT-IP), Wechsler Memory Scale-3rd Ed. (WMS-III): Spatial Span, Letter-Number Span (LNS), Hopkin’s Verbal Learning Test-Revised (HVLT-R), Brief Visuospatial Memory Test-Revised (BVMT-R), Neuropsychological Assessment Battery (NAB): Mazes, Mayer-Salovey-Caruso Emotional Intelligence Test (MSCEIT): Managing Emotions. Additional measures were administered as followed: Rey–Osterrieth Complex Figure Test (ROCFT) Copy^70^, Grooved Pegboard Test^71^, ROCFT Immediate Recall^69^, Pittsburgh Sleep Quality Index (PSQI)^72^, Beck Depression Inventory (BDI)^73^, Stroop Color Word Test (Stroop)^74^, the Positive and Negative Syndrome Scale (PANSS)^75^, Wechsler Test of Adult Reading (WTAR^76^, and the ROCFT Delayed Recall^70^.

The following neuropsychological measures were grouped into 12 cognitive domains for further analysis: (1) *Neurocognitive Composite Score*: BACS: Symbol Coding, Category Fluency: Animal Naming, Trail Making Test: Part A, CPT-IP, WMS-III: Spatial Span, LNS, HVLT-R, BVMT-R, NAB: Mazes, and MSCEIT: Managing Emotions; (2) *Speed of Information Processing*: BACS, Category Fluency: Animal Naming, Trail Making Test: Part A; (3) *Attention/vigilance:* CPT-IP; (4) *Working memory:* WMS-III, Spatial Span, LNS; (5) *Verbal learning:* HVLT-R; (6) *Visual learning:* BVMT-R; (7) *Reasoning and problem solving*: NAB: Mazes; (8) *Social cognition*: MSCEIT: Managing Emotions; (9) Visual Perceptual Delayed Recall: ROCFT Immediate and Delayed; (10) *Psychomotor Functioning:* The Groove Pegboard (dominant and non-dominant hands); (11) *Executive Functioning*: Trail Making Test A and B, Stroop; (12) *Abstract Thinking:* PANSS.

Raw data and Z-scores were transformed into T-scores by utilizing established normative data. Executive Functioning, Psychomotor Functions, and Abstract Thinking raw scores were calculated into T-scores based on the performance of controls (*N* = 16). Higher T-scores signified better performance across all measures.

### Data processing and analysis

All images were preprocessed by SPM12 software^77^ and Matlab 2019 (Mathworks Inc., Natick, MA). The raw EPI images were realigned to the mean of the time series to correct for head motion using the standard SPM12 routine. We used the “DRIFTER” toolbox^78^ for all rs-fMRI time-series to remove local oscillatory physiologic noise like cardiac and respiratory cycles. To account for whole brain influences we performed linear detrending. fMRI images were co-registered to the anatomical scans see Methods in^79^. The anatomical images were partitioned into gray matter, white matter and cerebrospinal fluid using SPM12’s “DARTEL” procedure^80^. Each participant's deformation map, obtained from the anatomical image, was applied to the functional images for normalization into the Montreal Neurological Institute (MNI) space with an isotropic voxel size of 2 mm^3^.

We used the “DPABI: Data Processing & Analysis” software package^81^ to calculate ALFF, f-ALFF and ReHo. In the software package, the time series was first converted to the frequency domain using a Fast Fourier Transform, and the averaged square root of the power spectrum for the predefined typical frequency interval 0.01–0.08 Hz was termed ALFF^81,82^. We applied a bandpass filter ranging from 0.01 to 0.08 Hz to all the ALFF and f-ALFF analyses. f-ALFF measures the power within the low frequency (0.01–0.08 Hz) divided by the total power in the entire detectable frequency range to represent the relative contribution of low frequency oscillations^55^. For ReHo we analyzed unsmoothed data as per DPABI recommendations^81^. We bandpass-filtered the data to 0.01–0.08 Hz and the ReHo cluster was for 27 voxels, along with smoothening the ReHo outcome (sm-ReHo) images by a 6 mm full-width-at-half-maximum Gaussian kernel similar to^81^. We inputted the *z* score signals (prefixed with z-ALFFmap, z-fALFFmap and szReHomap) outputted from DPABI^81^, for subsequent statistical analysis with SPM12 package^77^. Overlap in areas of difference of ALFF and ReHo indicates regions that are active at the specified frequency and are in sync with neighboring voxels, likely reflecting a large group of neurons firing together^55^.

Once we identified the brain regions showing significantly different ALFF or ReHo values compared to controls, we conducted additional correlation analysis between the pediatric HIV neurocognitive measures and average values for those regions.

### Statistical analyses

The Statistical Package for the Social Sciences (SPSS, V 24.0, IBM, Chicago, IL) was used to examine demographic and clinical parameters. Independent samples *t*-tests were performed to examine age, and gender differences between PHIV-infected and healthy control groups. Pearson's correlation was performed to examine the association between cognitive measures and functional connections in the PHIV-infected youth group. The significance level was set at *p* = 0.05.

We used Pearson’s correlation to inter-correlate each of the clinical parameters—scan CD4%, log viral load, along with their psychological performance metrics (IQ Neurocognitive score, Speed of Processing, Attention/Vigilance, Working Memory, Verbal Learning, Visual Learning, Reasoning and Problem Solving, Social Cognition, Overall Composite IQ score, Executive Functioning, Visual Perceptual Delayed Recall, Psychomotor Functions, Abstract Thinking), Maternal Substance Use, School Difficulties, and whether or not the PHIV had a diagnosis of HIV Encephalopathy. The significance level was set at *p* = 0.05. In order to avoid multicollinearity, we reported and removed from further analysis several psychological variables that were inter-correlated.

We used the SPM12 software package^77^ for ANCOVA analyses of control (n = 16) and PHIV (n = 11) groups with age and sex as co-variates. Traditional neuroimaging findings are reported as t-statistic, where a t statistic is calculated at each voxel location. Groups of adjacent voxels identified as significant are termed clusters. Clusters of rs-fMRI differences are overlaid on anatomical backgrounds for visualization. Correction for multiple comparisons was performed with cluster thresholding, which consists of two stages. After thresholding with an uncorrected threshold of *p* < 0.001 and minimum cluster size of 3, clusters are each thresholded based on family-wise error (FWE) correction at *p* < 0.05.

For the regions that showed significant differences in ALFF and ReHo between PHIV and healthy controls, we used intrinsic masking in SPM to correlate the ALFF and ReHo data in the 11 pediatric HIV patients with the clinical parameters of viral load, CD4 and neuropsychological variables. The significance level of contrasts was set to *p* = 0.001 with cluster size greater than or equal to 3.

## Results

The patient and healthy control groups’ demographic details are shown in Table 1; there were no significant differences in age or gender.

**Figure 1:**
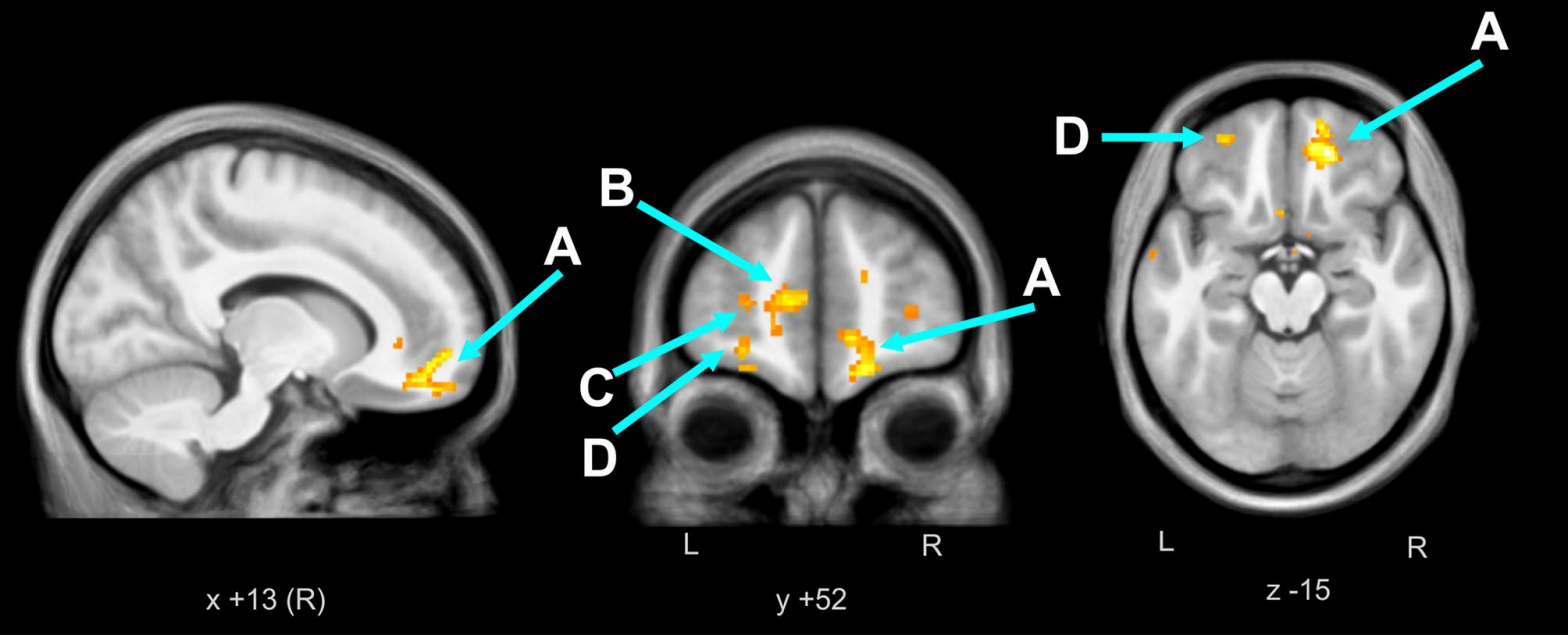
ALFF higher in pediatric HIV vs. Control. In pediatric HIV patients (n = 11) compared to controls (n = 16), we observed significantly higher cluster FWE-corrected *p* < 0.05 and uncorrected *p* < 0.05 values for the ALFF resting state fMRI activity in bilateral cerebral white matter. The regional activity was overlaid on anatomical mean of all 27 subjects’ T_1_ images. (**A**) Right cerebral white matter/right medial prefrontal cortex, (**B**) left cerebral white matter/left prefrontal cortex, (**C**) left cerebral white matter, (**D**) left cerebral white matter.

**Figure 2:**
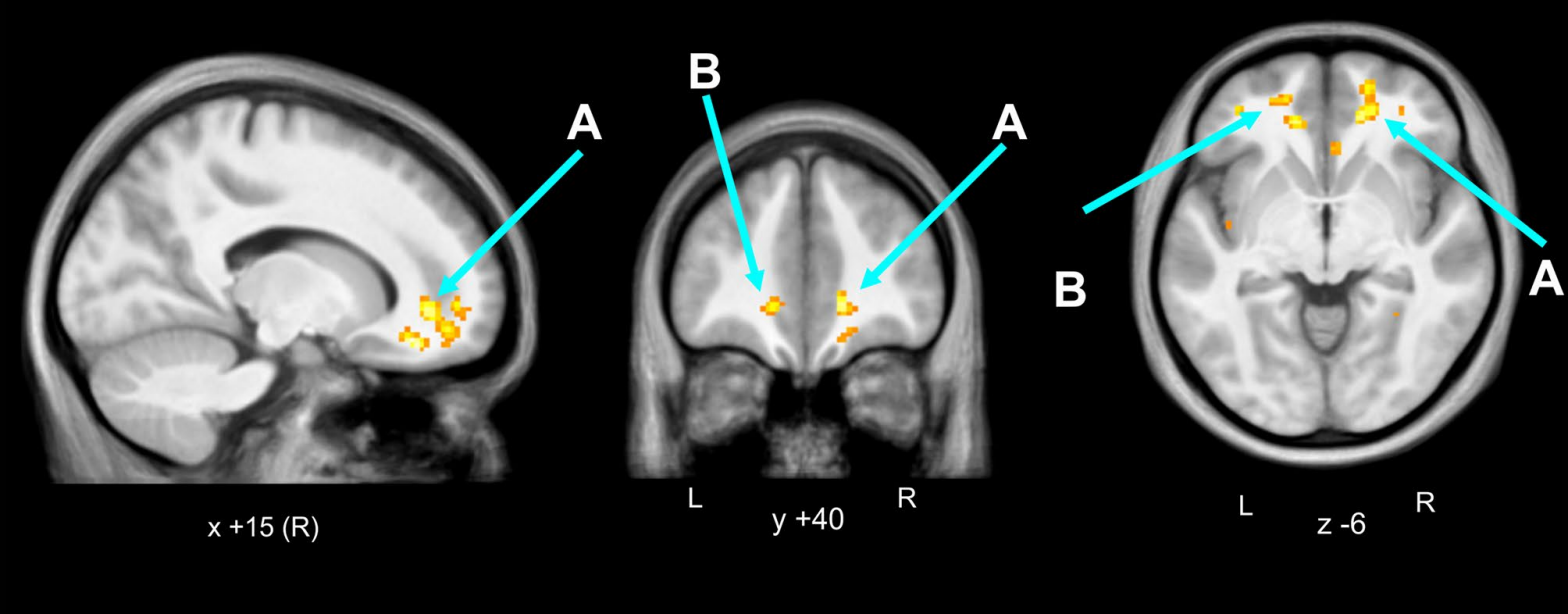
ReHo higher in pediatric HIV vs. Control. In pediatric HIV patients (n = 11) compared to controls (n = 16), we observed significantly higher cluster FWE-corrected *p* < 0.05 value and uncorrected *p* < 0.05 values for the ReHo resting state fMRI activity in bilateral cerebral white matter. The regional activity was overlaid on anatomical mean of all 27 subjects’ T_1_ images. (**A**) right cerebral white matter, (**B**) left cerebral white matter.

We found significantly higher ALFF and ReHo in the cerebral white matter in the medial orbital gyrus (or prefrontal cortex) for PHIV patients (n = 11) compared to controls (n = 16), with age and gender as co-variates. We overlaid clusters of ALFF and ReHo changes on average of the 27 subjects’ anatomical scans (Figs. 1 and 2). Table 2 depicts the brain regions showing higher ALFF and ReHo in PHIV patients compared to controls. We found predominantly in the bilateral cerebral white matter an increased spontaneous regional neuronal activity in PHIV compared to healthy controls. There were no brain regions that showed significantly lower ALFF or ReHo in PHIV compared to control. We did not obtain a significant difference in fALFF between the patients and controls.

**Table 2:** Resting fMRI data summary.

Type of resting fMRI data	Brain regions	Cluster p (FWE-corr)	Cluster p (unc)	Number of cluster voxels	MNI coordinate (mm) x	MNI coordinate (mm) y	MNI coordinate (mm) z	Peak *t*	Figure
ALFF	Right cerebral white matter/right medial prefrontal cortex	0.000	0.000	338	18	42	− 16	6.16	Figure 1A
	Left cerebral white matter/left prefrontal cortex	0.002	0.00	199	− 8	56	8	5.64	Figure 1B
	Left cerebral white matter	0.120	0.004	83	− 12	38	− 6	5.16	Figure 1C
	Left cerebral white matter	0.260	0.010	64	− 34	36	10	4.80	Figure 1D
ReHo	Right cerebral white matter	0.0005	0	264	16	34	− 16	6.11	Figure 2A
	Left cerebral white matter	0.8613	0.0411	39	− 12	38	− 6	5.36	Figure 2B

Brain regions with cluster FWE-corrected *p*  <  0.001 showing significantly higher regional neuronal activity (ALFF and ReHo) in patients with perinatally afflicted HIV (n = 11).

Table 3 shows positive (p < 0.01, cluster size ≥ 3*)* associations of log_10_ viral load with ALFF and ReHo for the 11 patients in regions of PHIV-Control differences (Table 1).

**Table 3:** Correlation of resting fMRI data with viral load.

Type of resting fMRI data	Brain Regions	Peak *p* (uncorr) and Type of correlation with log_10_ viral load	Number of cluster voxels	MNI coordinate (mm) x	MNI coordinate (mm) y	MNI coordinate (mm) x	Peak *t*	Figure
ALFF	Right cerebral white matter	0.001 (+ve)	6	12	34	− 2	4.24	Figure 1A
	Left cerebral white matter	0.002 (+ve)	3	− 36	34	16	3.97	Figure 1B
	Left cerebral white matter	0.004 (+ve)	8	− 44	44	− 2	3.35	Figure 1B
	Right cerebral white matter	0.004 (+ve)	3	16	40	− 6	3.32	Figure 1A
	Right cerebral white matter	0.006 (+ve)	3	16	36	− 14	3.12	Figure 1A
ReHo	Right cerebral white matter	0.002 (+ve)	4	16	32	− 16	3.82	Figure 2A

Brain regions masked with significantly higher regional neuronal activity in the pediatric HIV patients compared to healthy controls, showed positive correlation (peak uncorrected *p*  <  0.01, cluster size ≥ 3) of log_10_ viral load with regional neuronal activity in the 11 pediatric HIV patients. + ve indicate positive correlation and − ve indicate negative correlation.

Table 4 shows negative (p < 0.01, cluster size ≥ 3) associations of Social Cognition, Psychomotor Functioning and Abstract Thinking with ALFF at the left cerebral white matter in the left medial orbital gyrus and with the ReHo at the right cerebral white matter in the 11 PHIV patients in regions of PHIV-Control differences (Table 1). Table 4 also lists the only significantly positive (*p* < 0.01, cluster size ≥ 3) association of Social Cognition with ReHo, which appeared in the right central operculum/right cerebral white matter.

**Table 4:** Correlation of resting fMRI data with neurocognitive variables.

NP domains	Type of resting fMRI data	Brain regions	Peak *p* (uncorr)	Number of cluster voxels	MNI coordinate (mm) x	MNI coordinate (mm) y	MNI coordinate (mm) x	Peak *t*	Figure
Social cognition	ALFF	Left cerebral white matter/left medial orbital gyrus	0.000 (−ve)	3	22	48	− 18	6.02	Figure 1B
		Left cerebral white matter	0.001 (−ve)	3	− 50	4	− 12	4.26	Figure 1B
		Left cerebral white matter	0.002 (−ve)	7	− 32	36	20	3.84	Figure 1B
	ReHo	Right cerebral white matter	0.000 (−ve)	5	12	28	− 10	5.41	Figure 2A
	ReHo	Right central operculum /right cerebral white matter	0.002 (+ve)	4	48	0	− 4	3.71	Figure 2A
Psychomotor functions	ALFF	Right cerebral white matter/right medial orbital gyrus	0.002 (−ve)	4	18	40	− 18	3.97	Figure 1A
Abstract thinking	ALFF	Right cerebral white matter	0.001 (−ve)	3	14	56	− 16	4.77	Figure 1A
	ReHo	Right cerebral white matter	0.006 (−ve)	5	18	44	− 12	3.18	Figure 2A

Brain regions masked with significantly higher regional neuronal activity in the pediatric HIV group compared to healthy controls, showed mostly negative correlation (peak uncorrected *p*  <  0.01, cluster size ≥ 3) of Social Cognition, negative correlation of Psychomotor Functioning and negative correlation of Abstract Thinking with regional neuronal activity in the 11 pediatric HIV patients. + ve indicate positive correlation and − ve indicate negative correlation.

## Discussion

Youth perinatally infected with HIV showed altered resting state activity, reflecting differences in brain function relative to healthy counterparts. Specifically, we found higher activity of low frequency oscillations (ALFF) in PHIV youth compared to controls, especially in the cerebral matter of prefrontal cortex where it could indicate higher sympathetic activity. Per the original study by Biswal and colleagues, ALFF in a resting state reflects correlations between blood flow and oxygenation, which is interpreted as brain regions being functionally related^40^. Previous studies on acute traumatic brain injury reported higher ALFF and increased spontaneous activity in low frequency bands (0.01–0.08 Hz)^82^. We found a group of voxels in cerebral white matter in the medial orbital gyrus with higher ALFF together with a higher level of a marker of functional similarity, ReHo, in PHIV compared to controls. We did not observe a significant difference in the groups for in neural ALFF (f-ALFF); since this measure is considered gray matter specific^55^, the findings of altered ALFF likely reflect at least in part differences in non-neural physiology, including in the white matter; such fluctuations in the fMRI signal may reflect inflammation and glial activation in PHIV relative to the control group.

Global effects such as motion or cerebral blood flow changes are unlikely to have influenced the findings. Low-frequency fluctuations in white matter are reduced relative to grey matter by 60%^40^ and the significance of white matter spontaneous neuronal firing in resting fMRI data has not been reported previously for HIV adults or PHIV. We had removed physiological artifacts using DRIFTER toolbox^78^, and detrended the fMRI data with linear detrending tools (as in^40^). Additionally, we found that some ReHo and ALFF differences occurred in overlapping brain regions (cerebral white matter in the medial orbital gyrus). Thus, the findings of white matter differences in fMRI activity are unlikely to have been influenced by global effects.

Higher ALFF in particular could be related to underlying glial cycling or mitosis of glial cells. Microgliosis and neuroinflammation are long-term consequences of traumatic brain injury and pathogenesis in general^83^ and in PHIV we expect to find both microgliosis and neuroinflammation. HIV causes inflammation throughout the brain, which can persist despite control of the HIV virus in the peripheral blood^84^. The monocytes and T cells in the brain that are infected with HIV and have successfully crossed the blood–brain barrier can induce endothelial cells to release cytokines, consequently causing inflammation within the brain. In PHIV, this inflammation in the CNS may persist due to the difficulty of common medications being able to cross the blood–brain barrier into the CNS. The HIV-infected monocytes and T cells not only contaminate brain cells, but also release proinflammatory cytokines, viral proteins, and excitotoxins that can activate microglia, perivascular macrophages and astrocyte cells in the CNS and are potential reservoirs for the virus^85^. These are the main contributors to neuroinflammation in HIV infection and these cells release neurotoxic factors such as excitatory amino acids in addition to inflammatory mediators^86^. An HIV-infected CNS results in the increased activation of monocytes and macrophage, resulting in astrocytosis and microglial activation^87^. Glial cycling could also result from such pathophysiology, which could explain why we find higher ALFF activity in PHIV.

Our earlier finding of compromised white matter integrity found via Diffusion Tensor Imaging (DTI) in PHIV suggests structural differences may co-occur with the functional alterations, and inflammation is one possible cause of changes in both structure as seen with DTI^88^ and ALFF as seen here.

Recent research in adult HIV patients receiving cART vs healthy controls has found rs-fMRI differences mostly in ALFF measures^89–91^. In these studies, various regions showed increased or decreased magnitude of ALFF which differs from the findings in our study^89–92^. While there may be systematic differences between our PHIV group and other HIV populations, our small number of subjects precludes making strong generalizations.

The fact that we found higher ALFF in the cerebral white matter of orbital and frontal gyri in PHIV patients at the brain regions correlated with cognitive and emotional response could be indicative of ongoing neuroinflammatory insults^93,94^. Similar to our present study, studies of leukoaraiosis (LA) have found a higher ALFF in cerebral white matter of superior orbital frontal gyrus in the periventricular and subcortical areas of the brain. Moreover, LA patients also show cognitive impairment^95^ as found in our present study in the PHIV patients, suggesting there may be similar cognitive impairment and associated higher white matter ALFF activity and neuroinflammation in PHIV. Our findings are consistent with a previous study on postmortem brain tissue from patients with HIV-associated neurocognitive disorders, which showed signs of neuroinflammation^96^. It has been reported that some antiretroviral medications used to treat HIV can contribute to the likelihood of neurocognitive disorders^84^. Although new cART drugs are less toxic with fewer metabolic complications, chronic inflammation and other factors such as the irreparable damage of metabolic tissues suffered prior to the introduction of cART, side effects associated with other medications, and host genetic risk can still contribute to the neurocognitive impairment observed in PHIV-infected youth and in general HIV-infected patients.

Limitations of our study include the relatively small sample size, so further studies with larger cohorts are needed to confirm our findings. In addition, the cross-sectional design limited our ability to assess the impact of HIV on brain development over time. Future rs-fMRI study on PHIV youth should also include perinatally HIV-exposed uninfected youth apart from the HIV-unexposed healthy controls group for better distinguishing potential mechanisms.

## Conclusions

The findings are consistent with the hypothesis that long-term higher neuroinflammation and associated neurorepair in perinatally HIV-infected patients may be reflected in the higher regional spontaneous activity that we observe in the white matter in PHIV patients compared to healthy controls. Moreover, the higher cerebral white matter spontaneous activity correlated with higher viral load and decreased cognition, suggesting a role for neuroinflammation in impaired cognition. Resting state fMRI, particularly ALFF data that has been utilized to interpret neuroinflammation in this study, shows promise as a future tool to follow the effects of HIV on brain function, which is an important measure since these PHIV youth survive many years into adulthood. Such noninvasive measures may detect subtle ongoing inflammation, which could potentially be targeted with anti-inflammatory therapy or changes in antiretroviral treatment to preserve brain health in these surviving patients. In future, larger sample size studies should consider other neuroimaging techniques to confirm inflammation in the white matter in PHIV.
